# Large-Scale Screening and Identification of *S-RNase* Alleles in Chinese and European Apricot Accessions Reveal Their Diversity and Geographic Distribution Patterns

**DOI:** 10.3390/ijms26178667

**Published:** 2025-09-05

**Authors:** Junhuan Zhang, Meiling Zhang, Wenjian Yu, Fengchao Jiang, Li Yang, Juanjuan Ling, Haoyuan Sun

**Affiliations:** 1 Institute of Forestry and Pomology, Beijing Academy of Agriculture and Forestry Sciences, Beijing 100093, China; 2 Apricot Engineering and Technology Research Center, National Forestry and Grassland Administration, Beijing 100093, China; 3 Key Laboratory of Urban Agriculture (North China), Ministry of Agriculture and Rural Affairs, Beijing 100093, China

**Keywords:** apricot, self-(in)compatibility, *S-alleles*, geographic distribution

## Abstract

Apricot (*Prunus armeniaca* L.) exhibits a gametophytic self-incompatibility (GSI) system. To identify the *S*-genotypes of the main apricot cultivars, including 133 native Chinese cultivars and 35 foreign accessions, PCR was performed using a combination of five primers based on the conserved regions of *Prunus S-RNase* genes. After cloning and sequencing the PCR products, the *S*-genotypes of all 168 apricot cultivars were determined. A total of 46 different *S-RNase* alleles, with 15 new alleles, were identified. For all 168 accessions, the top five most frequent *S-alleles* were *S*_8_, *S*_11_, *S*_9_, *S*_16_, and *S*_53_. *S*_11_, *S*_8_, and *S*_16_ were the most frequent in Chinese cultivars, and *S*_9_, *S*_8_, and *S*_2_ were mostly found in European accessions. For Chinese apricot cultivars, the distribution of *S-alleles* among five geographic regions was also investigated. In Northwest China, *S*_16_ was the most frequent *S-allele*. In the Xinjiang region, *S*_66_, *S*_49_, and *S*_14_ were the top three most frequent *S-alleles*. In North China, *S*_8_, *S*_11_, and *S*_53_ were the top three most frequent *S-alleles*. In addition, the self-compatible type, *S_C_*, was not detected in these 133 Chinese accessions. Finally, the phylogenetic tree of apricot *S-alleles* indicated that there are four groups of *S-RNase* genes (*S*_97_/*S*_106_, *S*_14_/*S*_14*a*_/*S*_66_, *S*_9_/*S*_17_/*S*_44_, and *S*_23_/*S*_53_) presenting a very close relation. These results provide more data on the *S*-genotypes of apricot accessions, which can support future breeding programs by aiding in the selection of the appropriate parents and contributing to efficient orchard design by combining cultivars with suitable pollinizers.

## 1. Introduction

Apricot (*Prunus armeniaca* L.), one of the most popular temperate tree fruit species, is widely grown around the world. The total planting area of apricot trees worldwide reached 5.73 million acres, with a total production of about 4.48 million tons (FAOSTAT, 2023, https://www.fao.org/faostat/ (accessed on 19 August 2025)). As the primary center of origin for apricot (*Prunus armeniaca* L.), China has extensive apricot germplasm diversity, with over 2000 distinct cultivars and landraces. The apricot fruit is attractive due to its unique pleasant aroma and high nutritional value. Unfortunately, most Chinese cultivars exhibit self-incompatibility, resulting in low fruit setting [1]. In apricot production, the single plant yield has a significant positive correlation with the self-(in)compatibility of the cultivar [2]. Because of self-incompatibility, it is necessary to select and grow suitable pollinating trees when establishing an apricot orchard.

Traditional compatibility assessment in controlled pollination trials primarily relied on empirical knowledge derived from agricultural practice. Early diagnostic criteria classified cultivars as definitively self-compatible when exhibiting fruit set rates exceeding 6% through self-pollination [2,3]. However, the investigation of fruiting rates is time-consuming and labor-intensive. In the laboratory, fluorescence microscopy can be used to observe pollen tube growth, but this method requires expensive equipment and complex procedures. Advances in research have revealed that the self-incompatibility of apricots is gametophytic, controlled by a single *S*-locus with multiple alleles [4]. This locus includes at least the stigma *S-RNase* gene and the pollen *SFB* gene. When the pollen and stigma exhibit the same allele, the growth of the pollen tube in the stigma is hindered, resulting in self-incompatibility [5].

Studies have demonstrated that the *S-RNase* genes exhibit tissue-specific expression patterns in stigma tissues. *RNase* activity is crucial for the inhibition of pollen tube growth during the incompatibility response and may be involved in the degradation of ribosomal RNA [6,7]. Therefore, the *S*-genotype of a given cultivar can be quickly detected through specific PCR amplification of *S-alleles*, and the compatibility between any two cultivars can be determined by comparing their *S*-genotypes. This technology provides a scientific basis for the selection of pollen cultivars in production [8]. *S-allele* characterization has been successfully implemented across the main *Rosaceae* species, including apricot [9,10], plum [11,12], Japanese apricot [13], sweet cherry [14,15], almond [16], apple [17], and strawberry [18]. This methodology has also been extended to other self-incompatible fruit crops beyond Rosaceae, including citrus [19] and pomelo [20].

For apricot, extensive research has been conducted on the *S-RNase* genes of apricot worldwide since 1998. Burgos et al. used non-equilibrium pH gradient electrophoresis to separate and identify *S-RNases* associated with gametophytic self-incompatibility in nine apricot cultivars. This was the first study to report the *RNase* activities associated with the incompatibility alleles *S*_1_, *S*_2_, *S*_3_, *S*_4_, *S*_5_, and *S*_6_ and the compatibility *Sc* in apricot [4]. Subsequently, Romero cloned three *S-RNase* genes from the apricot genome [6]. In 2010, a total of 31 different *S*-genotypes were assigned to the 51 Turkish apricot cultivars. The *S-RNase* intron regions used to determine their lengths and the *S*-genotypes were detected via polymerase chain reaction (PCR) amplification [5]. The *S*-genotypes of 55 Moroccan apricot accessions were determined, resulting in 37 self-compatible genotypes [21]. The *S-alleles* of 44 new European apricot genotypes were further identified [22]. Boubakri et al. identified the *S*-genotypes of 68 Eurasian apricot variety groups from the Iran–Caucasus region and the Mediterranean basin planted in Tunisian regions. Self-compatible apricot cultivars were also discovered [23]. In contrast to Eurasian research, studies on Chinese apricot *S*-genotypes remain limited and have emerged more recently. In 2005, a pair of primers was designed, and *S-allele-specific* PCR was developed. Nine *S-alleles*, *S*_1_–*S*_9_, were first revealed via *S-allele*-specific PCR and confirmed via Southern blot analysis [24]. The *S*-genotypes of 16 apricot cultivars were also determined via the *S-allele* PCR approach, and the results were confirmed via cross-pollination tests among these cultivars [25]. Wu Jun et al. [26] analyzed the *S*-genotypes of 14 Chinese apricot cultivars and named eight new *S-alleles*. Jiang Xin et al. [27] detected the *S*-genotypes of 27 apricot varieties cultivated in Xinjiang and found 15 new *S-alleles*. Cumulatively, 96 apricot *S-RNase* genes have been registered in GenBank, reflecting both methodological progress and global collaboration.

However, despite the extensive documentation of apricot cultivars, research on their self-incompatibility (SI) systems remains disproportionately limited. Furthermore, the presence of synonymies and homonymies among some known *S-RNase* alleles led to a lack of comparability between different studies. Notably, identical cultivars have been assigned conflicting *S-RNase* genotypes in separate investigations. For instance, the *S-RNase* genotype of the same apricot cultivar ‘Yinxiangbai’ was reported as *S*_23_*S*_36_ by Wuyun et al. [28] vs. *S*_9_*S*_17_ by Zhang et al. [25]. ‘Honghebao’ was also given two distinct *S-RNase* genotypes, *S*_9_*S*_11_ and *S*_8_*S*_9_, in different studies [24,25], and the *S-RNase* genotype of ‘Xinshiji’ was recorded as *S*_7_*S*_8_ and *S*_9_*S*_10_ in different studies. These discrepancies have limited the exchange of information. As a result, systematic research on the molecular mechanisms underlying apricot self-incompatibility has been hindered.

In China, apricot cultivation spans extensive areas across distinct geographical regions, primarily taking place in North China (Beijing, Tianjin, Hebei, Shanxi), Northwest China (Gansu, Shaanxi), and Northeast China (Liaoning, Heilongjiang, Jilin). There are also supplementary cultivation zones in Shandong and Henan. Regional cultivars show strong locality-specific characteristics, and there is some varietal overlap between regions, which contributes to remarkably diverse germplasm. However, most cultivars exhibit self-incompatibility, with *S*-genotype characterization remaining incomplete for many cultivars. Current *S*-genotype data for Chinese apricots remains limited, with fewer than 70 cultivars documented to date [24,25,26]. This incomplete understanding of incompatibility relationships has impeded the use of parental selection in hybrid breeding programs and the configuration of effective pollination trees in commercial orchards.

In this study, the *S-RNase* genotypes of 168 apricot cultivars, primarily native to China, were determined through targeted PCR analysis and *S-RNase* sequencing. Then, the *S-allele* frequency distribution patterns in Chinese apricot accessions were compared to those in foreign ones, and the geographic distribution of *S-allele* frequencies within Chinese apricot cultivars was analyzed. Furthermore, we performed molecular characterization of self-compatibility determinants in selected high-yield genotypes, aiming to identify *S_C_* alleles within Chinese apricot accessions. The findings aid in establishing cross-incompatibility groups to avoid pollination problems in orchards and provide useful information for breeders in selecting parental genotypes. The novel *S-RNase* allele sequences obtained in this study provide critical data resources for advancing phylogenetic analyses of *S*-locus evolution within *Rosaceae* species.

## 2. Results

### 2.1. Identification of S-Alleles in Apricot

The *S*-genotypes of 168 apricot cultivars were characterized through PCR amplification of the second intron regions using five primer pairs, followed by sequencing and homology analysis with DNAMAN 8 software. Sequence alignment revealed 99–100% similarity among fragments of identical/near-identical length. Exon-derived amino acid sequences demonstrated complete conservation (100% homology) across all samples. Comprehensive analysis combining intron size polymorphisms with sequence patterns identified 46 distinct *S-alleles* through NCBI BLAST (https://blast.ncbi.nlm.nih.gov/Blast.cgi (accessed on 20 February 2025))verification. Thirty-one alleles matched previously reported apricot *S-RNase* genes, while fifteen represented novel *S-alleles*. The fragment sizes and the GenBank accession numbers are shown in Table 1. The second introns of all 31 *S-RNase* genes were found within the hypervariable region (RHV), with sizes ranging from 180 bp (*S*_10_) to 1749 bp (*S*_20_), demonstrating a high degree of length polymorphism that distinguishes the different *S-RNase* alleles.

Most alleles showed unique GenBank accession correspondence. However, some *S-RNase* genes, such as *S*_18_, *S*_40_, and *S*_52_, have the same *S-allele* accession number that corresponds to two GeneBank accession numbers. Furthermore, the gene sequences and amino acid sequences are also completely different. For *S*_52_, after BLAST searches in the NCBI database, the sequence of the *A-S*_52_ allele was matched to two *PaS*_52_-RNases from two different apricot cultivars, ‘Daguohuanna’ and ‘Kabakehuanna’, under the accession numbers KF951503.2 and HQ342882.1, respectively. KF951503.2 showed the complete sequence of *S*_52_, while HQ342882.1 was a partial sequence. In this study, the amino acid (AA) sequences from seven different cultivars with A-*S*_52_ showed the highest similarity to *S*_52_, with the accession number KF951503.2. It is different from the pattern of *S*_52_, and there were four cultivars that were assigned *S*_18_, associated with different Genebank accession Numbers: (DQ270000.1 and DQ870634.1) https://www.ncbi.nlm.nih.gov/nuccore/DQ270000.1/, https://www.ncbi.nlm.nih.gov/nuccore/DQ870634.1 (accessed on 20 February 2025)) are marked as *S*_18-1_ and *S*_18-2_ in this study. *S*_40-1_ and *S*_40-2_ represent two different *S*_40_ with different Genebank accession Nos. (GU354239.1 and HQ342870.1) (https://www.ncbi.nlm.nih.gov/nuccore/GU354239.1, https://www.ncbi.nlm.nih.gov/nuccore/HQ342870.1 (accessed on 20 February 2025)).

### 2.2. Identification of New S-Alleles in Apricot

**Figure 1:**
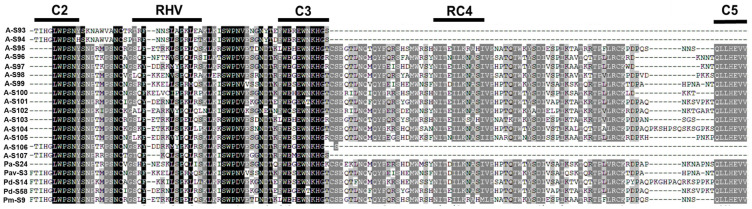
Alignment of the deduced amino acid sequences of *S-RNase* from apricot and other *Prunus* species. Four conserved regions, C2, C3, RC4 and C5, were marked with rectangles, and one hypervariable region, RHV, was underlined. A-: *S-RNases* of apricot (*P. armeniaca*) were detected in this study; Pa-: *S-RNases* of apricot (*P. armeniaca*) have been published; Pav-: *P. avium*; Pd-: *P. dulcis*; Pm-: *P. mume*. GenBank accession numbers of *Prunus* species: *Pa-S*_24_: ABS84176.1; *Pav-S*_3_: AAT72119.1; *Pd-S*_14_: CAJ77745.1; *Pd-S*_58_: CBI68346.1; *Pm-S*_9_: BAF91157.1. The different colors represent the conserved percentage among sequences: black, 100%; darkgrey, 80%; grey, 60%.

The DNA sequences were initially aligned with the *Prunus S-RNase* cDNA sequence exhibiting the highest homology from GenBank. Intron/exon boundaries were identified using the conserved GT/AG splicing rule, with intron sizes determined subsequently. Corresponding amino acid sequences were deduced through DNAMAN software analysis and compared against existing *S-RNase* homologs in GenBank. Fifteen novel *S-alleles* were identified with characteristic *Prunus S-RNase* structural features, including four conserved domains (C2, C3, RC4, and C5), a hypervariable region (RHV), and an undocumented, unique intron size configuration. Critical analysis revealed distinct RHV variations in these sequences compared to all registered *P. armeniaca S-RNase* genes in GenBank. Based on sequence homology analysis through NCBI GenBank and following the nomenclature protocols of Vilanova et al. [29] and Halázs et al. [5], these new alleles were designated as *S*_93_–*S*_107_, continuing the existing *S-RNase* gene number in GenBank. The novel sequences have been deposited in GenBank under the accession numbers PV206780–PV206794, with specific assignments corresponding to each allele (Table 2). The second introns within the RHV demonstrated significant length polymorphism (90 bp -1214 bp), providing distinctive molecular characters for different *S-alleles* (Figure 1 and Table 2).

### 2.3. Identification of Sc-Allele

**Figure 2:**
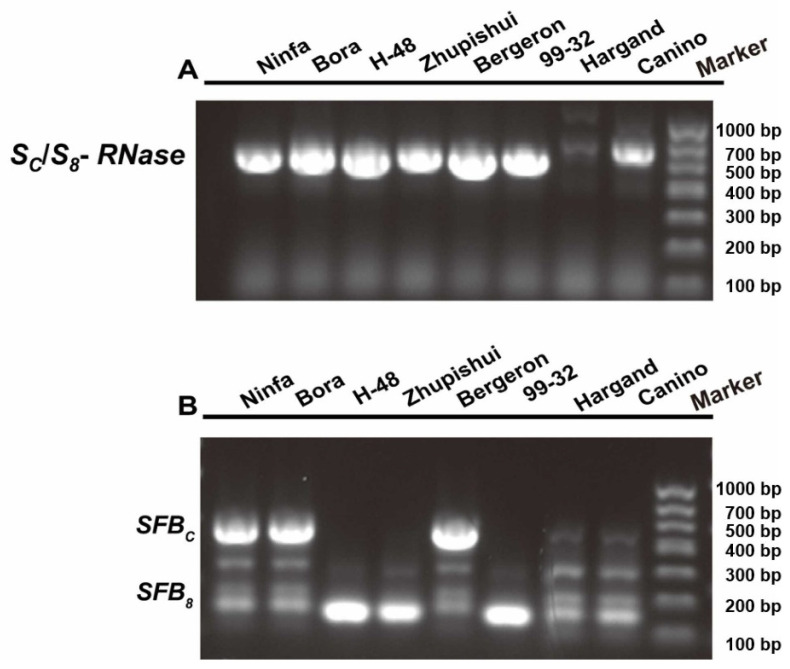
PCR detection and reliable differentiation of *S_C_*- and *S*_8_-haplotypes in eight apricots. (**A**) PCR used to identify selectively the *S*_8_/*S_C_*-RNase alleles. (**B**) Amplification of the *SFB* gene used to differentiate between *SFB_C_* and *SFB*_8_ alleles [M = 1 kb + DNA ladder].

Previous studies have confirmed that the coding regions of *S*_8_- and *S_C_*-RNase alleles are identical, with the *S*_8_- and *S_C_*-haplotypes differing exclusively in their SFB gene structure. Specifically, a 358 bp insertion was identified in the *SFB_C_*. To discriminate the *S_C_*-haplotype, we implemented a two-tiered molecular strategy. Initially, the allele-specific primer pair AprSC8R/PaConsI F was selected to amplify the *S_C_*/*S*_8_-RNase allele. As demonstrated in Figure 2A, a 546 bp fragment was successfully amplified in six cultivars, including the positive control cultivars ‘Bergeron’ (*S*_2_*S_C_*) and ‘Bora’ (*S*_9_*S_C_*), with determined *S*-genotypes [30]. In contrast, no amplification products were observed in the negative control cultivar ‘Hargrand’ (*S*_1_*S*_2_). Subsequently, employing the primer pair AprFBC8 [5], we distinguished between the *S*_8_- and *S_C_*-alleles. Cultivars carrying the *SFB_C_*-allele showed an amplification product fragment of approximately 500 bp, whereas those with the *SFB*_8_-allele produced a fragment of about 150 bp (Figure 2B). The combinatorial results conclusively revealed that only three cultivars, ‘Nifa’, ‘Bora’, and ‘Bergeron’, were self-compatible, carrying the *S_C_*-haplotype. The *S*_8_-allele was identified in ‘H-48’, ‘Zhupishui’, and ‘99-31’ germplasm (Figure 2B).

### 2.4. Analysis of S-Genotypes of 168 Apricot Cultivars

Table 3 presents the *S*-genotype profiles of 168 apricot cultivars collected from diverse geographical regions. Among these cultivars, 122 (72.6%) accessions exhibited heterozygous *S*-genotypes at their *S-RNase* loci, demonstrating two distinct *S-alleles* for each cultivar. The other 46 cultivars, such as ‘Dabada’, ‘Fangshanhongxing’, ‘Guanyelian’, ‘Liquanerzhuanzi’, ‘Jingren No.1’, ‘Zhupishui’, ‘Daxingmei’, and ‘99-45’, showed mono-allelic expression at the *S*-locus, with only one detectable *S-allele*, while the complementary allele remained unidentified. Notably, 31 cultivars were found to carry novel *S-RNase* alleles (*S*_93_–*S*_107_) that were previously uncharacterized.

These results were supported by a previous study on controlled cross-pollination tests for some apricot cultivars [1]. The fruit set percentages of ‘luotuohuang’ × ‘Honghebao’, ‘luotuohuang’ × ‘Dapiantou’, ‘Dapiantou’ × ‘Honghebao’, ‘Xinong 25’ × ‘Luotuohuang’ were 10.8–16.7%. According to the accepted criteria [2,3], these cultivars are cross-compatible. Correspondingly, in this study, each combination of cultivars has a different *S*-genotype. The S-genotypes of ‘luotuohuang’, ‘Honghebao’, ‘Dapiantou’, and ‘Xinong 25’ were *S*_8_*S*_11_, *S*_9_*S*_16_, *S*_36_*S*_102_, and *S*_10_*S*_36_, respectively. ‘Chuanling’ (*S*_8_*S*_53_) and ‘Luotuohuang’ (*S*_8_*S*_11_), which shared one *S-allele*, were considered as semi-compatible and, usually, cannot be selected as pollinizers for each other.

### 2.5. S-Allele Frequency Distribution Patterns Between Chinese and Foreign Apricot Accessions

**Figure 3:**
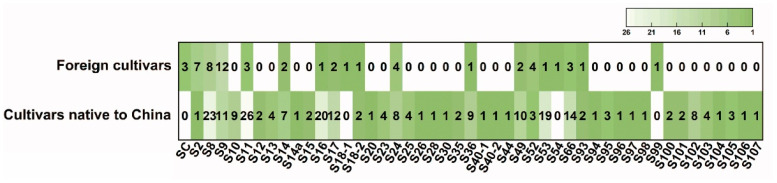
*S-allele* frequency distribution of Chinese apricots and foreign accessions.

As illustrated in Figure 3, *S*_8_ emerged as the predominant *S-allele* across all 168 apricot accessions, followed sequentially by *S*_11_, *S*_9_, *S*_16_, and *S*_53_. Comparative analysis revealed distinct distribution patterns between Chinese cultivars and foreign accessions. For Chinese apricot cultivars, *S*_11_ was the most frequent *S-allele* (occurred in 26 genotypes), followed by *S*_8_ (in 23 genotypes), *S*_16_ (in 20 genotypes), *S*_53_ (in 19 genotypes), *S*_66_ (in 14 genotypes), *S*_17_ (in 12 genotypes), *S*_9_ (in 11 genotypes), and *S*_49_ (in 10 genotypes). The remaining 35 *S-alleles* occurred at relatively lower frequencies, each present in fewer than 10 genotypes. For *S*_2_, *S*_14*a*_, *S*_20_, *S*_26_, *S*_28_, *S*_30_, *S*_40-1_, *S*_40-2_, *S*_44_, *S*_94_, *S*_96_, *S*_97_, *S*_98_ *S*_104_, *S*_106_, and *S*_107_, each *S-allele* was detected in only one genotype. For foreign accessions, *S*_9_, *S*_8_, *S*_2_, *S*_24_, and *S*_52_ were the top three most frequent, occurring in 12, 8, and 7 genotypes, respectively. Each of the eight *S-alleles*, including *S*_16_, *S*_18-1_, *S*_18-2_, *S*_36_, *S*_53_, *S*_54_, *S*_93_, and *S*_99_, was also detected in only one European cultivar. In both Chinese apricot cultivars and foreign accessions, *S*_8_ and *S*_9_ were the relatively more frequent *S-alleles*. *Sc* was found in only three European genotypes, and could not be detected in the tested Chinese apricot cultivars. In addition, it was found that *S*_53_ was mostly found in white-fleshed apricot cultivars, such as ‘Xiaoyubada’, ‘Fangshanxiangbai’, ‘Shanbaixing’, ‘Chuanling’, and ‘Zaoxiangbai’, which comprised 11 of the 19 cultivars with *S*_53_ (Table S1).

### 2.6. Geographic Distribution Patterns of S-Allele Frequencies in Chinese Apricot Cultivars

**Figure 4:**
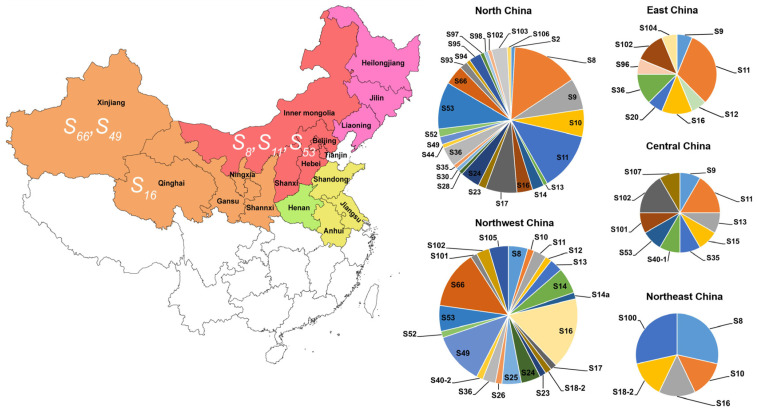
*S-allele* frequency distribution according to geographic areas in China. A map of the five major production areas of apricots in China: northwest area (orange color), North China (pink color), northeast area (red color), Central China (green color), and East China (yellow color). Relative frequencies for *S-alleles* (pie charts) are shown for each area.

The distribution of *S-alleles* demonstrated significant geographic dependency among Chinese apricot accessions, with distinct frequency patterns emerging across five major regions (Figure 4 and Table S2). The key distribution characteristics of the alleles exhibit a diverse geographic spectrum. There are three alleles, *S*_11_, *S*_16_, and *S*_102_, that are present in four regions. Additionally, there are tri-regional alleles, such as *S*_8_, *S*_9_, *S*_10_, *S*_13_, *S*_18_, *S*_36_, and *S*_53_; bi-regional alleles, like *S*_14_ *S*_17_, *S*_23_, *S*_24_, *S*_35_, *S*_40_, *S*_49_, *S*_52_, *S*_66_, and *S*_101_; and 25 alleles that are specific to single regions. In terms of regional frequency profiles, the northwestern region of China, encompassing Gansu, Shaanxi, Ningxia, and Qinghai, has 40 accessions containing 23 *S-alleles*, with *S*_16_ being the dominant allele. The Xinjiang region has 18 accessions with 14 alleles, with *S*_66_ leading in frequency at 25%, followed by *S*_49_ at 22%, and *S*_14_ at 11% (Table 3). In North China, which includes Beijing, Tianjin, Hebei, and Shanxi, there are 63 accessions exhibiting 28 alleles. The most frequent alleles are *S*_8_ at 15%, *S*_11_ at 13%, *S*_53_ at 10%, and *S*_9_ and *S*_17_ at 7%. In the northeastern part of China, there are five cultivars with five *S-alleles* (*S*_8_, *S*_10_, *S*_16_, *S*_18-2_, and *S*_100_) detected, with *S*_8_ and *S*_100_ being the dominant *S-alleles*. In Central China, specifically Henan Province, there are seven accessions containing 11 alleles, with *S*_11_ and *S*_102_ co-dominant at 17% each. Lastly, in East China, which covers Shandong and Anhui, there are ten accessions with nine alleles, with *S*_11_ being the predominant allele at 31%.

### 2.7. S-RNase Gene Sequence Alignment and Phylogeny

**Figure 5:**
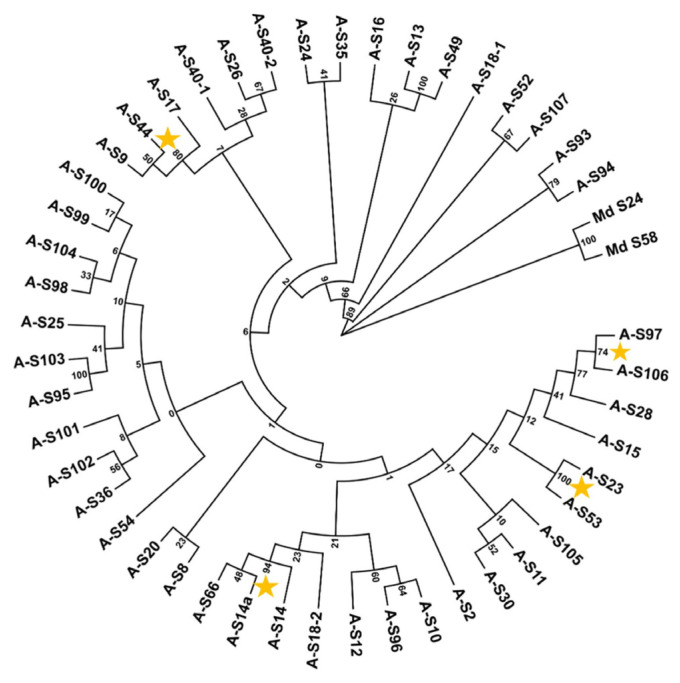
Phylogram depicting evolutionary relationships among apricot *S-RNase* alleles with apple (*Malus domestica*). *S-RNase* alleles (GenBank accessions: AWL24801.1, *Md-S*_24_; AWL24810.1, *Md-S*_58_) used as outgroup. Yellow asterisk: the groups of *S-RNase* have a close relation.

The predicted amino acid sequences between the C2 and C5 regions from 46 detected *S-RNases* were aligned with each other using the Clustal W algorithm, and a neighbor-joining tree was constructed. The phylogenetic tree demonstrated that there are four groups of *S-RNase* genes that are closely related, such as *S*_97_ and *S*_106_; *S*_14_, *S*_14*a*_, and *S*_66_; *S*_9_, *S*_17_, and *S*_44_; and *S*_23_ and *S*_53_ (Figure 5). Detailed sequence comparisons showed distinct amino acid (AA) variations among these groups. For *S*_97_ and *S*_106_, a single amino acid difference was observed in the C3 domain. *S*_14*a*_ vs. *S*_14_, displayed two AA and four AAs variations in the C2 and C3 conserved regions, respectively. Both *S*_14_ and *S*_14*a*_ exhibited an additional valine residue (V) in the hypervariable region (RHV) compared to *S*_66_. Compared to *S*_9_, *S*_17_ lacked one valine residue (V) in the RHV, while *S*_44_ lacked two amino acid residues (leucine and valine, L and V). In comparison with *S*_53_, *S*_23_ has a deletion of one amino acid (a tyrosine, Y) in the RHV region and has another difference of one amino acid in the C3 region.

## 3. Discussion

The spatial distribution of *S-RNase* alleles exhibits distinct biogeographical clustering, serving as a molecular signature for tracing germplasm evolution. Our analyses revealed pronounced regional specificity. *S*_66_ and *S*_49_ mainly appear in the Xinjiang apricot population. *S*_16_ is present with high frequency in the north regions, including Northwest China (Figure 4 and Table S2). These differences may reflect varying environmental selection pressures or the effects of genetic drift across regions. Data from the literature indicated that the *S*_7_-allele is only present in Southern Europe and North Africa [30]. The alleles *S*_10_–*S*_14_ showed an Armenian origin and have also been detected in Turkish and Moroccan apricots, but are absent in Western and Southern European countries [23]. The self-compatible type, *S_C_*, was not detected in Chinese apricots, only existing in European apricot cultivars. It is generally accepted that the genetic diversity of apricots decreases from east to southwest, and in this context, it is questionable whether *S_C_* might be one of the causes [30]. In addition, in this study, we also found that *S*_8_, *S*_9_, and *S*_11_ appear at relatively high frequencies in both Chinese native cultivars and some European cultivars (Figure 3), which might be ancient genes of apricot cultivars.

In plum, the *S*-locus genotype is suitable for diversity studies in polyploid *Prunus* species [12]. Alburquerque et al. [31] declared that the number of *S-alleles* in apricot should be low, as only eight alleles were detected in Mediterranean and North American accessions. Halász et al. [10] identified more (at least nine) new alleles in the tested Eastern European and Central Asian genotypes, and further explained that the Central Asian eco-geographical group has a more variable genetic background compared to the European group. In this study, 43 *S-alleles* were detected among 133 Chinese apricot cultivars, and the diversity of *S-alleles* is related to the rich genetic diversity of Chinese apricot resources.

The *S*-genotype may be highly associated with certain trait characteristics of the cultivar. In this study, we found that *S*_53_ appears at a high frequency in white-fleshed cultivars (Table S1). Cultivars with the *S*_8_ genotype have a higher yield, similar to that of *S_C_* cultivars. The self-compatibility of *S*_8_ cultivars needs further verification (Table S3). *S*_66_ mainly appears in the Xinjiang apricot population. A defining morphological feature of these apricot cultivars is their glabrous exocarp (fruit epidermis), characterized by a smooth cuticular structure and distinct glossiness (Table S4). For botanical classification, they are exclusively classified as *Prunus armeniaca* var. glabra Sun S.X. Supporting this point, Wu et al. [26] reported that the more frequent occurrence of these three alleles may be due to their linkage to beneficial traits or conferring adaptation to local environmental conditions. Also, in sweet cherry, certain *S-alleles* have a higher selective advantage and confer beneficial economic characteristics [32].

The *S-allele* type has been used as a means of cultivar identification. Among these tested cultivars, ‘Jingzaohong’ (*S*_9_*S*_36_) was a new accession developed by cross-breeding in recent years. Its female parent and pollen parent were ‘Dapiantou’ (*S*_36_*S*_102_ and ‘Honghebao’ (*S*_9_*S*_26_), respectively. ‘*S*_9_’ and ‘*S*_36_’ were inherited from ‘Honghebao’ (*S*_9_*S*_16_) and ‘Dapiantou’ (*S*_36_*S*_102_), respectively (Table 3). The present data shows good correspondence between the *S-alleles* of the parents and those inherited by the individual cultivars. Another new apricot cultivar ‘Jingluofeng (*S*_11_*S*_102_)’ was selected from the seeding of the cultivar ‘Luotuohuang (*S*_8_*S*_11_)’, and ‘*S*_11_’ was inherited from its female parent ‘Luotuohuang’ (*S*_8_*S*_11_). These results are from the previous report by Zhang et al. on Chinese apricots [25]. For the cultivars ‘Hongfeng’ (*S*_9_*S*_10_) and ‘Xinshiji’ (*S*_9_*S*_10_), ‘*S*_9_’ and ‘*S*_10_’ were inherited from their parents ‘Honghebao’ (*S*_9_*S*_11_) and ‘Erhuacao’ (*S*_10_*S*_11_), respectively. Common alleles may indicate a common origin [10], a notion supported by SSR markers [33].

In order to verify the homonymy in *S-RNase* naming among apricot cultivars, we analyzed the *S-alleles* identified in this study with all known synonyms from previous studies (Table S5). There are 17 cultivars in this study that were assigned *S*-genotypes in previous studies. Some cultivars, such as ‘Jiguang’, ‘Zhanggongyuan’, and ‘Bergeron’, had the same *S*-genotypes in this study as in previous studies, suggesting that the cultivar names are accurate. ‘Qiaoerpang’, ‘Yinxiangbai’, ‘Honghebao’, ‘Canino’, and ‘Ninfa’ had only one similar *S-allele*. The other nine cultivars presented completely different *S*-genotypes. These cultivars may be regarded as instances of homonymy.

China is recognized as the center of apricot origin and has an extremely abundant apricot germplasm [34]; however, few *S*-genotypes of this germplasm have been determined. In this study, *S*-genotypes of as many as 133 apricots native to China were identified. However, there were 46 cultivars that exhibited only one *S-allele* (Table 3). In this experiment, all 46 varieties were analyzed using five pairs of primers, and each primer pair consistently yielded only a single allele. A similar result was reported by Boubakri et al. in a study on Tunisian apricot cultivars, in which only one *S-allele* was detected [23]. However, the underlying reasons for this observation remain unclear. One possibility is that large intron fragments within the *S-RNase* of these cultivars may hinder effective amplification [26], making the current primers less suitable for these specific genotypes. Additionally, some cultivars have complex genetic backgrounds. For instance, ‘Jingren No.2’, ‘Jingren No.1’, ‘Jingren No.3’, ‘Jingren No.4’, and ‘Jingren No.5’ were all derived from distant hybridization between apricot (*Prunus armeniaca*) and almond (*Prunus amygdalus*) [35]. Although the primers used are generally applicable across *Prunus* species, designing primers based on specific sequence features and screening optimal primer combinations may improve amplification efficiency in these genetically complex accessions. Another plausible explanation is that some cultivars exhibit homozygosity at the S-locus, a phenomenon previously reported in *Prunus* species [10]. To accurately determine the *S*-haplotypes of these undetected alleles, more advanced genomic approaches such as long-read sequencing should be employed.

Currently, comprehensive *S*-genotype data can provide scientific guidance for apricot production. First, *S*-genotype data enables the creation of empirically validated cross-incompatibility matrices for optimized orchard pollinizer design. These cross-compatibility matrices, which group cultivars by *S*-genotype, directly support pollinizer selection (Table S6). Secondly, this data provides a molecular foundation for strategic parental selection in apricot breeding programs, particularly for developing self-compatible cultivars through specific *S-alleles*. The newly identified *S-RNase* genes substantially expand the known allele diversity within *P. armeniaca* and provide new molecular markers for phylogenetic studies of *S*-locus evolution in *Rosaceae*.

## 4. Materials and Methods

### 4.1. Plant Materials

A total of 168 apricot accessions with known and unknown compatibility phenotypes were analyzed in this study. The collection comprised 133 Chinese cultivars and 35 international accessions, including 32 European cultivars, 1 American accession, and 2 Japanese genotypes. These cultivars were obtained from the apricot germplasm collection of the Institute of Forestry and Pomology, Beijing Academy of Agriculture and Forestry Sciences.

### 4.2. DNA Extraction

Total genomic DNA was extracted from young leaves using the Hi-DNAsecure Plant kit DP350-03 (Tiangen Biotech, Beijing, China) according to the manufacturer’s instructions. The concentration of the isolated DNA was determined using a Thermo Scientific NanoDrop™ spectrophotometer (Thermo Fisher Scientific, Waltham, MA, USA.) and via electrophoresis on 1% agarose gels.

### 4.3. PCR Amplification

Five primer pairs, previously reported as universal primer combinations for *Prunus* plants, were used to perform the specific PCR amplification of *S-alleles*: EM-PC2consFD+EM-PC3consRD, PruC2+Amy-C5R, PruC2+PCE-R, As1II+AmyC5R, and PaConsII-F+PaConsII-R. The specific primer sequences are shown in Table 4. PCR cycling parameters and conditions were as described in the respective references.

### 4.4. Cloning and Sequencing of S-Alleles

The PCR-amplified fragments were excised from 1.2% agarose gels and purified using the Agarose Gel DNA Purification Kit (TaKaRa, Dalian, China). The purified products were cloned into the pEASY-Blunt Simple Cloning vector (Tiangen Biotech, Beijing, China) following the manufacturer’s instructions and transformed into *Escherichia coli* DH5α. To obtain an accurate sequence and avoid errors caused by PCR, three independent positive clones of each fragment were sequenced by Sangon Biotech Company (Shanghai, China).

To identify the *S_C_*-haplotype, a two-step approach was used, as described by Halász et al. [5]. For the first step, an allele-specific reverse primer, AprSC8-R, was used in combination with PaConsI-F [14] to amplify the *S_C_*/*S*_8_-RNase allele. For the second step, specific primers, AprFBC8-F and AprFBC8-R, were designed selectively to amplify the SFBC/8 alleles [38].

### 4.5. Analysis for Sequence Data and Identification of S-Alleles

DNAMAN 8 software was employed for multiple sequence alignment and annotation of putative *S-alleles*. Nucleotide sequences were subjected to homology analysis using BLASTN against the NCBI nucleotide database. Intron–exon boundaries were determined through comparative alignment of genomic DNA with corresponding DNA references from *Prunus armeniaca S*-alleles. The translated amino acid sequences spanning the conserved C2–C5 domains were derived from the annotated nucleotide data. Subsequent protein-level verification employed BLASTP (https://blast.ncbi.nlm.nih.gov/Blast.cgi (accessed on 20 February 2025)) to compare deduced amino acid sequences against the NCBI non-redundant database.

### 4.6. Construction of Phylogenetic Tree Based on S-RNase Gene Sequences

All the predicted amino acid sequences between the C2 and C5 regions from 46 detected *S-RNase* genes were aligned with each other using the ClustalW algorithm in MEGA 11, and a neighbor-joining tree was constructed. Two apple (*Malus domestica*) *S-RNase* alleles (*Md-S*_24_: AWL24801.1; *Md-S*_58_: AWL24810.1) were used as outgroup. The Poisson correction method was used to compute evolutionary distances, and the reliability test was performed 1000 times using Bootstrap.

## 5. Conclusions

In this study, the *S*-genotypes of 168 apricot cultivars were determined via cloning and sequencing the specific PCR products. A total of 46 different *S-RNase* alleles, with 31 previously reported and 15 new alleles, were identified. The self-compatible type, *S_C_*, was not detected in the 133 Chinese accessions tested. Then, the *S-allele* frequency distribution patterns were investigated, and the results indicated that *S*_8_ emerged as the predominant *S-allele* across all tested apricot accessions, followed sequentially by *S*_11_, *S*_9_, *S*_16_, and *S*_53_. The geographic distribution patterns of *S-allele* frequencies in Chinese apricot cultivars were also analyzed. The most frequent alleles in Northern China are *S*_8_, *S*_11_, and *S*_53_. In the northwestern region of China, *S*_16_ was the dominant *S-RNase* gene. Based on the *S-RNase* gene sequence data, the phylogenetic tree of apricot *S-alleles* was constructed. These results can benefit future breeding programs by aiding the selection of appropriate parents and can contribute to efficient orchard design by promoting the planting of cross-compatible apricot cultivars.

## Figures and Tables

**Table 1:** Sequence from 168 apricot cultivars aligned with 31 published *S-alleles*.

*S-Allele*	PCR Fragment Size (bp)/Intron Sizes (bp)					Genebank Accession No.
	EM-PC2consFD,	Pru-C2,	Pru-C2,	ASI II,	PaCons II-F,	
	EM-PC3consRD	Amy-C5	PCE-R	Amy-C5	PaCons II-R	
*S*_2_	895/706	1096/706				AY587562.1
*S*_8_		827/410	573/409	928/411		AY884212.1
*S*_9_		885/467				AY853594.1
*S*_10_	266/180					AY846872.1
*S*_11_	464/275	672/275				DQ868316.1
*S*_12_	359/171					DQ870628.1
*S*_13_	401/212					DQ870629.1
*S*_14_	493/305					DQ870630.1
*S*_14*a*_	495/309					GU574199.1
*S*_15_	469/283					DQ870631.1
*S*_16_	481/292	700/292				DQ870631.1
*S*_17_	657/461					DQ270001.1
*S*_18-1_ *	307/108					DQ270000.1
*S*_18-2_ *	1337/1148	1546/1148				DQ870634.1
*S*_20_	1936/1749					EF160078.1
*S*_23_	693/505					EU037262.1
*S*_24_	357/168	588/168				EU037263.1
*S*_25_	772/583	994/584				EU037264.1
*S*_26_			416/289			EU037265.1
*S*_28_		1352/946				EU836684.1
*S*_30_		726/285				EF185301.1
*S*_35_	312/124					GU574196.1
*S*_36_		718/299				GU574198.1
*S*_40-1_ *	539/353	749/353				GU354239.1
*S*_40-2_ *				542/164		HQ342870.1
*S*_44_			635/464			HQ342874.1
*S*_49_		653/212				HQ342879.1
*S*_52_	1296/1111	1512/1110				KF951503.2
*S*_53_		965/508				KF975455.2
*S*_54_					1296/891	KT223013.1
*S*_66_		704/308				JQ317152.1

* *S*_18-1_ and *S*_18-2_, represent two different *S*_18_ alleles with different Genebank accession No.; *S*_40-1_ and *S*_40-2_ represent two different *S*_40_ alleles with different Genebank accession No.

**Table 2:** Fifteen new *S-alleles* in apricot accessions.

*S-Alleles*	Cultivar No.	Cultivar Name	Primer Pairs and PCR Fragment Size (bp)/Intron Sizes (bp)		GeneBank Accession No.
			EM-PC2consFD, EM-PC3consRD	Pru-C2, Amy-C5	
*S*_93_	22	Jingjia No. 2	273/90		PV206781
*S*_94_	46	Dafeng	502/319		PV206782
*S*_95_	28	Jingren No.4		683/275	PV206783
*S*_96_	74	Hongjinzhen		575/173	PV206784
*S*_97_	58	Xingtaihongjiexing		746/458	PV206785
*S*_98_	25	Jingren No.1		871/460	PV206791
*S*_99_	158	Harmat		884/460	PV206786
*S*_100_	82	Dongning No.2		924/522	PV206792
*S*_101_	96	Lintonghongxing		1348/928	PV206787
*S*_102_	23	Jingluofeng		1416/996	PV206793
*S*_103_	52	Longwangmao		1452/1044	PV206788
*S*_104_	75	Jinkaite		1466/1035	PV206789
*S*_105_	100	Niujiaobangzi		1625/1214	PV206794
*S*_106_	31	Longquanwuxiangbai	533/346		PV206790
*S*_107_	69	Yuhankui	1227/1046		PV206780

**Table 3:** *S*-genotypes of 168 apricot cultivars.

No.	Cultivar	Province, Country of Origin	*S*-Genotype	Areas
1	Baixing 10-38	Beijing, China	*S*_9_ *S*_10_	North China
2	Beianhe	Beijing, China	*S*_9_ *S*_17_	
3	Beishandabian	Beijing, China	*S*_10_ *S*_53_	
4	Beizhaihongxing	Beijing, China	*S*_93_ *S*_103_	
5	Chuanling	Beijing, China	*S*_8_ *S*_53_	
6	Dabada	Beijing, China	*S*_36_	
7	Fangshanhongxing	Beijing, China	*S*_11_	
8	Fangshanxiangbai	Beijing, China	*S*_17_ *S*_53_	
9	Guajiayutianhexiangbai	Beijing, China	*S*_8_ *S*_17_	
10	H20-5	Beijing, China	*S*_9_ *S*_95_	
11	H21-25	Beijing, China	*S*_23_ *S*_53_	
12	H23-37	Beijing, China	*S*_14_ *S*_66_	
13	H23-43	Beijing, China	*S*_14_ *S*_66_	
14	H23-44	Beijing, China	*S*_14_ *S*_66_	
15	H-48	Beijing, China	*S*_8_ *S*_52_	
16	Honghuomeizi	Beijing, China	*S*_8_ *S*_53_	
17	Huangjianzui	Beijing, China	*S*_2_ *S*_66_	
18	Jingcuihong	Beijing, China	*S*_10_ *S*_11_	
19	Jingfeihong	Beijing, China	*S*_8_ *S*_11_	
20	P35-146	Beijing, China	*S*_8_ *S*_30_	
21	Jingjia No.1	Beijing, China	*S*_24_ *S*_49_	
22	Jingjia No.2	Beijing, China	*S*_24_ *S*_93_	
23	Jingluofeng	Beijing, China	*S*_11_ *S*_102_	
24	Jingluohong	Beijing, China	*S*_8_ *S*_95_	
25	Jingren No.1	Beijing, China	*S*_98_	
26	Jingren No.2	Beijing, China	*S*_8_	
27	Jingren No.3	Beijing, China	*S*_103_	
28	Jingren No.4	Beijing, China	*S*_95_	
29	Jingren No.5	Beijing, China	*S*_24_	
30	Jingxianghong	Beijing, China	*S*_10_ *S*_11_	
31	Jingzaohong	Beijing, China	*S*_9_ *S*_36_	
31	Longquanwuxiangbai	Beijing, China	*S*_53_ *S*_106_	
33	Luotuohuang	Beijing, China	*S*_8_ *S*_11_	
34	Mituoluo	Beijing, China	*S*_11_	
35	P51-54	Beijing, China	*S*_11_ *S*_17_	
36	Pingguohong	Beijing, China	*S*_8_ *S*_66_	
37	Shanbaixing	Beijing, China	*S*_17_ *S*_53_	
38	Shanhuangxing	Beijing, China	*S*_8_ *S*_11_	
39	Xiaoyubada	Beijing, China	*S*_23_ *S*_53_	
40	Yingchun	Beijing, China	*S*_24_ *S*_36_	
41	Zaoxiangbai	Beijing, China	*S*_10_ *S*_53_	
42	Zhuyaozi	Beijing, China	*S*_10_ *S*_35_	
43	Guanlaoyelian	Tianjin province, China	*S*_8_ *S*_16_	
44	Wanxiangbai	Tianjin province, China	*S*_17_ *S*_53_	
45	Cangzaotian No.1	Hebei province, China	*S*_11_ *S*_49_	
46	Chuanzhihong	Hebei province, China	*S*_8_ *S*_24_	
46	Dafeng	Hebei province, China	*S*_8_ *S*_94_	
48	Erhongxing	Hebei province, China	*S*_11_ *S*_17_	
49	Ganyu	Hebei province, China	*S*_8_ *S*_16_	
50	Jiguang	Hebei province, China	*S*_8_ *S*_9_	
51	Jinyu	Hebei province, China	*S*_13_ *S*_52_	
52	Longwangmao	Hebei province, China	*S*_11_ *S*_103_	
53	Muguaxing	Hebei province, China	*S*_11_ *S*_16_	
54	Qingmisha	Hebei province, China	*S*_9_ *S*_44_	
55	Shizixing	Hebei province, China	*S*_10_ *S*_53_	
56	Tianedan	Hebei province, China	*S*_9_ *S*_16_	
57	Xingtaidahongxing	Hebei province, China	*S*_17_ *S*_36_	
58	Xingtaihongjiexing	Hebei province, China	*S*_8_ *S*_97_	
59	You No.1	Hebei province, China	*S*_11_ *S*_103_	
60	You No.2	Hebei province, China	*S*_11_	
61	Zaohongxing	Hebei province, China	*S*_36_	
62	Zaohuang	Hebei province, China	*S*_9_ *S*_53_	
63	Guanyelian	Shanxi province, China	*S*_28_	
64	Hongbada	Henan province, China	*S*_101_	Central China
65	Lixing	Henan province, China	*S*_40-1_	
66	Mixiangxing	Henan province, China	*S*_11_ *S*_15_	
67	Yangshaohuang No.1	Henan province, China	*S*_13_ *S*_102_	
68	Yangshaohuang No.2	Henan province, China	*S*_36_ *S*_102_	
69	Yuhankui	Henan province, China	*S*_11_ *S*_107_	
70	Yuzaoguan	Henan province, China	*S*_9_ *S*_53_	
71	Badou	Anhui, China	*S*_36_ *S*_102_	East China
72	Caizihuang	Shandong province, China	*S*_11_ *S*_16_	
73	Honghebao	Shandong province, China	*S*_9_ *S*_16_	
74	Hongjinzhen	Shandong province, China	*S*_96_	
75	Jinkaite	Shandong province, China	*S*_11_ *S*_104_	
76	Kuijin	Shandong province, China	*S*_11_ *S*_102_	
77	Laoshanhong	Shandong province, China	*S*_11_	
78	Pingdingzhen	Shandong province, China	*S*_12_ *S*_36_	
79	Qingdaodahong	Shandong province, China	*S*_11_	
80	Zaoyu	Shandong province, China	*S*_20_	
81	Dongning No.1	Heilongjiang province, China	*S*_16_ *S*_100_	Northeast China
82	Dongning No.2	Heilongjiang province, China	*S*_100_	
83	Baixing	Liaoning province, China	*S*_10_	
84	Daxingmei	Liaoning province, China	*S*_8_	
85	Guofeng	Liaoning province, China	*S*_8_ *S*_18-2_	
86	Caoxing	Gansu province, China	*S*_8_ *S*_17_	Northwest China
87	Dajiexing	Gansu province, China	*S*_16_	
88	Dapiantou	Gansu province, China	*S*_36_ *S*_102_	
89	Zhupishui	Gansu province, China	*S*_8_	
90	Taoxing	Ningxia province, China	*S*_16_	
91	Meixing	Qinghai province, China	*S*_25_	
92	Caopixing	Shaanxi province, China	*S*_16_	
93	Haidongxing	Shaanxi province, China	*S*_16_	
94	Jidanxing	Shaanxi province, China	*S*_25_ *S*_26_	
95	Lanzhuhong	Shaanxi province, China	*S*_16_ *S*_23_	
96	Lingtonghongxing	Shaanxi province, China	*S*_101_	
97	Lintonghongxing No.2	Shaanxi province, China	*S*_16_	
98	Liquanerzhuanzi	Shaanxi province, China	*S*_16_	
99	Machuanling	Shaanxi province, China	*S*_11_ *S*_16_	
100	Niujiaobangzi	Shaanxi province, China	*S*_105_	
101	Niujiaohuang	Shaanxi province, China	*S*_105_	
102	Qinwang	Shaanxi province, China	*S*_40-2_	
103	Touwojie	Shaanxi province, China	*S*_16_	
104	Xinong 25	Shaanxi province, China	*S*_10_ *S*_36_	
105	Yinxiangbai	Shaanxi province, China	*S*_36_ *S*_53_	
106	Zaotianhe	Shaanxi province, China	*S*_16_ *S*_105_	
107	Zhanggongyuan	Shaanxi province, China	*S*_24_ *S*_25_	
108	Ake	Xinjiang, China	*S*_12_ *S*_66_	
109	Chibangzi	Xinjiang, China	*S*_13_ *S*_49_	
110	Cuijianali	Xinjiang, China	*S*_49_ *S*_66_	
111	Dabaiyou	Xinjiang, China	*S*_18-2_ *S*_49_	
112	Daguohuanna	Xinjiang, China	*S*_14_ *S*_49_	
113	Dayoujia	Xinjiang, China	*S*_49_ *S*_66_	
114	Heiyexing	Xinjiang, China	*S*_16_ *S*_66_	
115	Kezimayisang	Xinjiang, China	*S*_14*a*_ *S*_66_	
116	Kuikepiman	Xinjiang, China	*S*_11_ *S*_53_	
117	Kumaiti	Xinjiang, China	*S*_49_ *S*_66_	
118	Liguangxing	Xinjiang, China	*S*_24_ *S*_49_	
119	Muyage	Xinjiang, China	*S*_14_ *S*_66_	
120	Pinaizi	Xinjiang, China	*S*_13_ *S*_49_	
121	Qiaoerpang	Xinjiang, China	*S*_14_ *S*_66_	
122	Saimaiti	Xinjiang, China	*S*_24_ *S*_53_	
123	Shushangganxing	Xinjiang, China	*S*_14_ *S*_66_	
124	Xinjiangshaxing	Xinjiang, China	*S*_8_ *S*_102_	
125	Xinshisheng	Xinjiang, China	*S*_52_ *S*_53_	
126	Bingtangwei	China	*S*_17_ *S*_25_	Unclear
127	Haihongzhen	China	*S*_9_ *S*_17_	
128	Haiquanhong	China	*S*_8_ *S*_11_	
129	Hongxing	China	*S*_11_	
130	Kuhehonglian	China	*S*_16_ *S*_102_	
131	Longjingbaixing	China	*S*_15_ *S*_16_	
131	Xiaopuxiangbai	China	*S*_17_ *S*_53_	
133	Yinxing	China	*S*_23_ *S*_53_	
134	Meiwuming	American	*S*_2_ *S*_8_	Foreign areas
135	99-2	Czech Republic	*S*_8_ *S*_9_	
136	99-12	Czech Republic	*S*_24_	
137	99-15	Czech Republic	*S*_8_ *S*_9_	
138	99-27	Czech Republic	*S*_52_	
139	99-31	Czech Republic	*S*_8_ *S*_66_	
140	99-37	Czech Republic	*S*_17_ *S*_18-2_	
141	99-38	Czech Republic	*S*_11_	
142	99-43	Czech Republic	*S*_9_ *S*_17_	
143	99-44	Czech Republic	*S*_24_ *S*_9_	
144	99-45	Czech Republic	*S*_11_	
145	Aurora	Czech Republic	*S*_8_ *S*_9_	
146	Betinka	Czech Republic	*S*_8_ *S*_52_	
147	Hargand	Czech Republic	*S*_2_	
148	Jennycot	Czech Republic	*S*_2_ *S*_9_	
149	Jitka	Czech Republic	*S*_24_ *S*_49_	
150	LE5137	Czech Republic	*S*_24_	
151	Rumjanaja	Czech Republic	*S*_8_ *S*_53_	
152	Bergeron	France	*S*_2_ *S_C_*	
153	Canino	France	*S*_2_ *S*_9_	
154	Early orange	France	*S*_9_ *S*_11_	
155	Cegledi bibor kajszi	Hungary	*S*_14_ *S*_66_	
156	Cegledi orias	Hungary	*S*_14_ *S*_66_	
157	Cegledi piroska	Hungary	*S*_36_	
158	Harmat	Hungary	*S*_99_	
159	B088	Italy	*S*_54_	
160	B089	Italy	*S*_2_ *S*_9_	
161	B095	Italy	*S*_49_ *S*_93_	
162	Bora	Italy	*S*_9_ *S_C_*	
163	Corlate	Italy	*S*_18-1_	
164	Ninfa	Italy	*S*_2_ *S_C_*	
165	Wondercot	Italy	*S*_9_ *S*_52_	
166	Yidalixing	Italy	*S*_52_	
167	Pinghexing	Japan	*S*_8_ *S*_9_	
168	Xinzhoudashi	Japan	*S*_16_	

**Table 4:** Sequences of five primer pairs used for *S-RNase* gene amplification.

Number	Primer Name	Sequence (5′ to 3′)	References
1	EM-PC2consFD	TCACM * ATYCATGGCCTATGG	Sutherland et al., 2004 [36]
	EM-PC3consRD	AW * CTR * CCRTGY * TTGTTCCATTC	
2	Pru-C2	CTATGGCCAAGTAATTATTCAAACC	Tao et al., 1999 [37]
	Pru-C5	TACCACTTCATGTAACAACTGAG	
3	Pru-C2	CTATGGCCAAGTAATTATTCAAACC	Tao et al., 1999; Wu et al., 2009 [26,37]
	PCE-R	TGTTTGTTCCATTCGCCTTCCC	
4	AS1II	TATTTTCAATTTGTGCAATGG	Tamura et al., 2000 [16]
	AmyC5R	CAAAATACCACTTCATGTAACAAC	
5	PaCons II-F	GGCCAAGTAATTATTCAAACC	Sonneveld et al., 2003 [14]
	PaCons II-R	CATAACAAARTACCACTTCATGTAAC	

* M = A/C; Y = C/T; W = A/T; and R = A/G.

## Data Availability

The data is contained within the article and Supplementary Materials.

## Supplementary Materials

The following supporting information can be downloaded at: https://www.mdpi.com/article/10.3390/ijms26178667/s1.
