# Supplementary figures and images for: Large-Scale Screening and Identification of S-RNase Alleles in Chinese and European Apricot Accessions Reveal Their Diversity and Geographic Distribution Patterns
Source: Int J Mol Sci. 2025 Sep 5;26(17):8667. doi: 10.3390/ijms26178667 (PMC12428848; doi:10.3390/ijms26178667)

## Slide 1
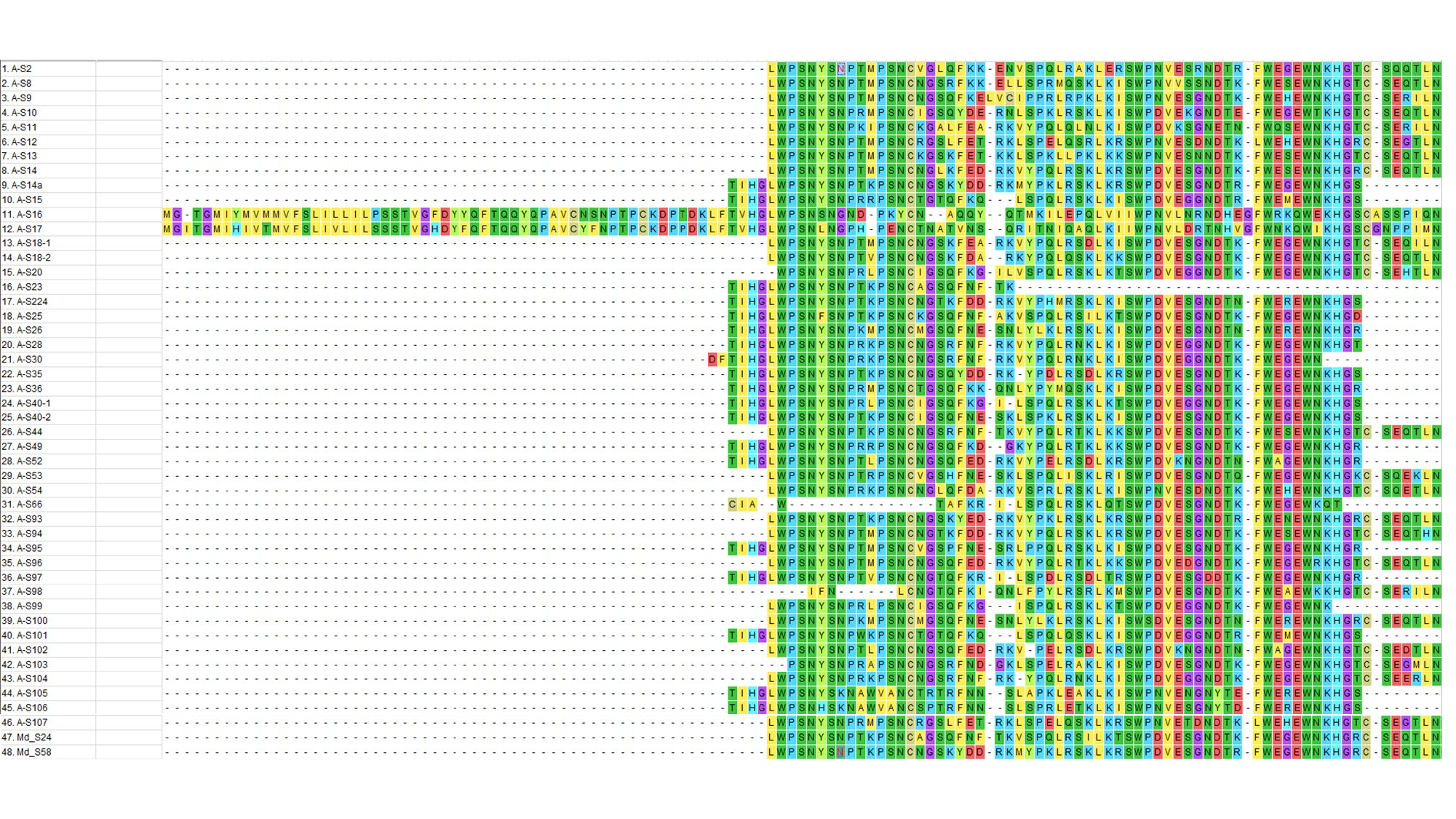

## Slide 2
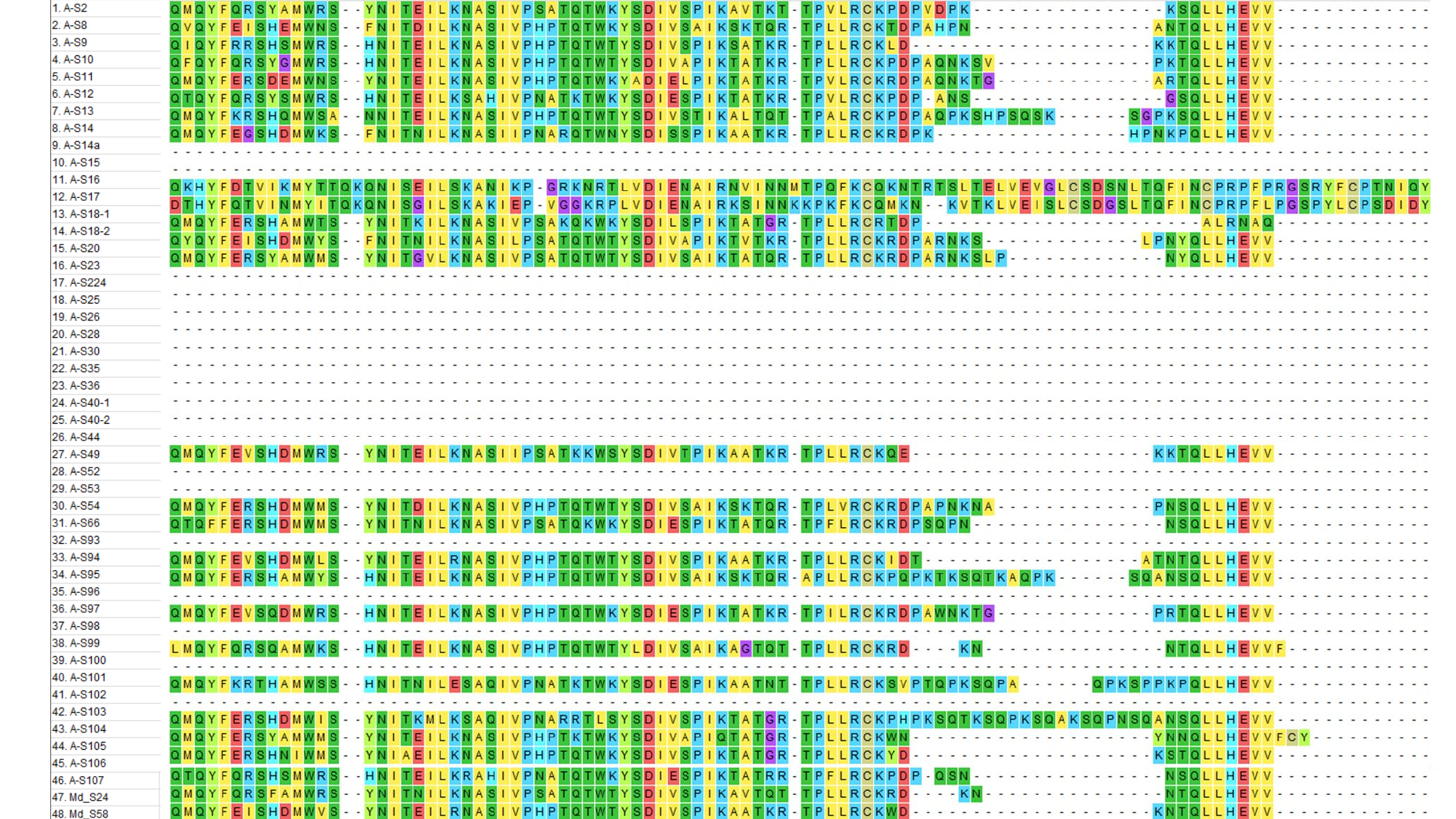

Supplement: Supplementary file 1 [file ijms-26-08667-s001.zip › ijms-3793935-Supplementary Materials-proof/full sequence aligment.pptx]
